# Supplementary material for: ADAM9 promotes type I interferon-mediated innate immunity during encephalomyocarditis virus infection
Source: Nat Commun. 2024 May 16;15:4153. doi: 10.1038/s41467-024-48524-6 (PMC11098812; doi:10.1038/s41467-024-48524-6)
Supplement: Supplementary file 2 — Reporting Summary [file 41467_2024_48524_MOESM2_ESM.pdf]

nature portfolio

Corresponding author(s): Evelyn A. Kurt-Jones, Ph.D.

Last updated by author(s): Feb 16, 2024

Reporting Summary

Nature Portfolio wishes to improve the reproducibility of the work that we publish. This form provides structure for consistency and transparency in reporting. For further information on Nature Portfolio policies, see our Editorial Policies and the Editorial Policy Checklist.

Statistics

For all statistical analyses, confirm that the following items are present in the figure legend, table legend, main text, or Methods section.

n/a	Confirmed	
☐	☒	The exact sample size (*n*) for each experimental group/condition, given as a discrete number and unit of measurement
☐	☒	A statement on whether measurements were taken from distinct samples or whether the same sample was measured repeatedly
☐	☒	The statistical test(s) used AND whether they are one- or two-sided<br>*Only common tests should be described solely by name; describe more complex techniques in the Methods section.*
☒	☐	A description of all covariates tested
☒	☐	A description of any assumptions or corrections, such as tests of normality and adjustment for multiple comparisons
☐	☒	A full description of the statistical parameters including central tendency (e.g. means) or other basic estimates (e.g. regression coefficient) AND variation (e.g. standard deviation) or associated estimates of uncertainty (e.g. confidence intervals)
☐	☒	For null hypothesis testing, the test statistic (e.g. *F*, *t*, *r*) with confidence intervals, effect sizes, degrees of freedom and *P* value noted<br>*Give P values as exact values whenever suitable.*
☒	☐	For Bayesian analysis, information on the choice of priors and Markov chain Monte Carlo settings
☒	☐	For hierarchical and complex designs, identification of the appropriate level for tests and full reporting of outcomes
☒	☐	Estimates of effect sizes (e.g. Cohen's *d*, Pearson's *r*), indicating how they were calculated

Our web collection on statistics for biologists contains articles on many of the points above.

Software and code

Policy information about availability of computer code

Data collection	No software was used for data collection.
Data analysis	GraphPad Prism software (version 9.0; GraphPad, San Diego, CA) was used for data analysis

For manuscripts utilizing custom algorithms or software that are central to the research but not yet described in published literature, software must be made available to editors and reviewers. We strongly encourage code deposition in a community repository (e.g. GitHub). See the Nature Portfolio guidelines for submitting code & software for further information.

Data

Policy information about availability of data

All manuscripts must include a data availability statement. This statement should provide the following information, where applicable:

- Accession codes, unique identifiers, or web links for publicly available datasets
- A description of any restrictions on data availability
- For clinical datasets or third party data, please ensure that the statement adheres to our policy

All supporting data have been provided in the Source Document.

Research involving human participants, their data, or biological material

Policy information about studies with human participants or human data. See also policy information about sex, gender (identity/presentation), and sexual orientation and race, ethnicity and racism.

Reporting on sex and gender	N/A
Reporting on race, ethnicity, or other socially relevant groupings	N/A
Population characteristics	N/A
Recruitment	N/A
Ethics oversight	N/A

Note that full information on the approval of the study protocol must also be provided in the manuscript.

Field-specific reporting

Please select the one below that is the best fit for your research. If you are not sure, read the appropriate sections before making your selection.

☒ Life sciences ☐ Behavioural & social sciences ☐ Ecological, evolutionary & environmental sciences

For a reference copy of the document with all sections, see nature.com/documents/nr-reporting-summary-flat.pdf

Life sciences study design

All studies must disclose on these points even when the disclosure is negative.

Sample size	Sample sizes for experiments were informed by consultation with a biostatistician and validated in prior research indicating that n = 3-7 biological replicates were sufficient for detecting significant differences with group and time as factors and include an interaction term to test differences in trend over time between the groups. Simulations were created using Stata 16.1 (StataCorp, LLC College Station TX) and used 2000 simulations for the estimate of power.<br>The number of experiments and replicates was based on standard practices and previous peer-reviewed publications (PMID: 33727702, PMCID: PMC8103894; PMID: 30723129 PMCID: PMC6428755). In general, two or three independent experiments were conducted with three biological replicates or technical replicates where applicable. Details for each experiment are included in the respective figure legend.
Data exclusions	No data were excluded
Replication	All experiments were replicated two to three times.
Randomization	Cages of age-matched mice were of each genotype and were randomized to groups. Experimental groups were assigned in a semi-randomized manner to ensure a balanced distribution of gender across treatments. Samples were processed in parallel to minimize variability.
Blinding	IAll experiments and data analyses were completed by an experimenter blinded to group allocation during data collection.

Reporting for specific materials, systems and methods

We require information from authors about some types of materials, experimental systems and methods used in many studies. Here, indicate whether each material, system or method listed is relevant to your study. If you are not sure if a list item applies to your research, read the appropriate section before selecting a response.

Materials & experimental systems

n/a	Involved in the study	
☐	☒	Antibodies
☐	☒	Eukaryotic cell lines
☒	☐	Palaeontology and archaeology
☐	☒	Animals and other organisms
☒	☐	Clinical data
☒	☐	Dual use research of concern
☒	☐	Plants

Methods

n/a	Involved in the study	
☒	☐	ChIP-seq
☒	☐	Flow cytometry
☒	☐	MRI-based neuroimaging

Antibodies

Antibodies used	Antibodies to detect cardiac troponin I (cTNI, Life diagnostics), IFN-β (PBL Assay Science), and IL-6 (R&D) were used to perform ELISAs according to the manufacturer's instructions. Anti-ADAM9 (CST, Cat# 2099S, 1:100) for IP; anti-ADAM9 (Bio-Techne Corporation, Cat# AF949, 1:1,000) for IB; anti-ADAM10 (Proteintech, Cat# 25900-1-AP, 1:1,000); anti-ADAM12 (Proteintech, Cat# 14139-1-AP, 1:1,000); anti-ADAM17 (Proteintech, Cat# 29948-1-AP, 1:1,000); anti-MDA5 (CST, Cat# 5321, 1:1,000); anti-MAVS (Santa Cruz, Cat# sc-365333, C-1, 1:500); anti-HA (CST, Cat# 3724, 1:1,000); anti-ACTIN (GeneTex, Cat# GTX629630, GT5512, 1:2,000); Normal Rabbit IgG (CST, Cat# 2729, 1 mg ml-1); Anti-TNNT (DSHB, Cat# RV-C2, 1:50); PEI (Polysciences, Cat# 00618); Lipofectamine2000 (Thermo Fisher, Cat# 11668019); Dynabeads Protein G (Invitrogen, Cat# 10009D); anti-FLAG M2 beads (Sigma-Aldrich, Cat# M8823); protease inhibitor cocktail (Sigma-Aldrich, Cat# P2714).
Validation	All antibodies were purchased from commercial vendors who have validated the antibodies for the use of Western blot and Co-immunoprecipitation.

Eukaryotic cell lines

Policy information about cell lines and Sex and Gender in Research

Cell line source(s)	HeLa, Vero E6, BHK-21, HEK293T, and Hepa1-6 cells were obtained from the American Type Culture Collection (ATCC).
Authentication	All cell lines were obtained from ATCC, which performs validation of their cell lines as part of their quality control process, including STR and mycoplasma testing.
Mycoplasma contamination	All cell lines were tested for mycoplasma contamination every 3–4 months and were negative for mycoplasma contamination.
Commonly misidentified lines (See ICLAC register)	None of the cell lines used are listed under the ICLAC commonly misidentified cell lines.

Animals and other research organisms

Policy information about studies involving animals; ARRIVE guidelines recommended for reporting animal research, and Sex and Gender in Research

Laboratory animals	The study used male and female C57BL/6 and ADAM9 KO mice (PMID: 11839819) ages 4–6 weeks old. Mice were housed at no more than 4 animals per cage and were separated by gender. Mice had free access to food and water. The animals were on a 12:12 light/dark cycle, with a room temperature of 700F +/- 20F and humidity of 30-70%.
Wild animals	No wild animals were used in the study.
Reporting on sex	Both sexes were included. No differences were observed between sexes.
Field-collected samples	No field collected samples were used in the study.
Ethics oversight	All experimental procedures conducted on mice were approved by the UMass Chan Institutional Animal Care and Use Committee.

Note that full information on the approval of the study protocol must also be provided in the manuscript.

Plants

Seed stocks	N/A
Novel plant genotypes	N/A
Authentication	N/A
